# An Open-Label, Phase II Study of the Safety of Pirfenidone in Patients with Idiopathic Pulmonary Fibrosis (PIPF-002)

**DOI:** 10.1007/s41030-018-0053-y

**Published:** 2018-06-01

**Authors:** Mark H. Gotfried, Carlos E. Girod, Danielle Antin-Ozerkis, Tracy Burgess, Indiana Strombom, John L. Stauffer, Klaus-Uwe Kirchgaessler, Maria L. Padilla

**Affiliations:** 1 Pulmonary Associates, Phoenix, AZ USA; 2 The University of Texas Southwestern Medical Center, Dallas, TX USA; 3 Yale School of Medicine, New Haven, CT USA; 4 Genentech, Inc., South San Francisco, CA USA; 5 F. Hoffmann-La Roche Ltd., Basel, Switzerland; 6 Icahn School of Medicine at Mount Sinai, New York, NY USA

**Keywords:** IPF, Pirfenidone, Safety

## Abstract

**Introduction:**

PIPF-002 was a phase 2, multicenter, open-label study of pirfenidone in patients with idiopathic pulmonary fibrosis (IPF) or other types of pulmonary fibrosis (PF). PIPF-002 terminated after pirfenidone became commercially available in the United States. The goal of PIPF-002 was to characterize the long-term safety of pirfenidone in these patients.

**Methods:**

Between August 2003 and September 2006, 83 patients (IPF: 81, PF: 2) enrolled. Patients received pirfenidone in three divided doses daily, with the maintenance dose and schedule determined by enrollment group assignment. Treatment continued until patient withdrawal or study termination (2015). Treatment-emergent adverse events (TEAEs) were assessed by descriptive statistics.

**Results:**

At baseline, median age was 70 years, mean percent predicted forced vital capacity was 67.7%, 33.7% of patients had cardiac disorders, 51.8% had gastroesophageal reflux disease, and 63.9% were receiving concomitant prednisone. Median pirfenidone dose and exposure duration were 2400 mg/day and 3.0 years, respectively. Cumulative total exposure was 279.7 patient-exposure years (PEY). Most patients (98.8%) reported ≥ 1 TEAE, with an overall incidence rate of 460.5 per 100 PEY. The most frequent TEAEs (incidence rate per 100 PEY) were nausea (23.6), IPF progression (16.1), fatigue (11.8), dyspnea (11.4), upper respiratory tract infection (11.4), and cough (10.7). Serious TEAEs were reported in 49 patients; the most frequent serious TEAEs were IPF progression and pneumonia. The most common reason for discontinuation was TEAEs (35 patients; 12.5 patients per 100 PEY), most frequently IPF progression and nausea. Overall, 21 patients died (7.5 per 100 PEY); 16 deaths were IPF-related.

**Conclusions:**

Long-term safety and tolerability of pirfenidone findings in this study were consistent with the known safety profile of pirfenidone; no new safety signals were identified. These data support the continued use of pirfenidone in patients with IPF.

**Funding:**

F. Hoffmann-La Roche Ltd./Genentech, Inc.

**Trial Registration:**

ClinicalTrials.gov identifier, NCT00080223.

**Plain Language Summary:**

Plain language summary available for this article.

**Electronic supplementary material:**

The online version of this article (10.1007/s41030-018-0053-y) contains supplementary material, which is available to authorized users.

## Plain Language Summary

Idiopathic pulmonary fibrosis (IPF) is a progressive, irreversible, and fatal lung disease. Pirfenidone is a medication that slows disease progression in patients with IPF. This study evaluated the long-term safety of pirfenidone in 81 patients with IPF and in two patients with other types of pulmonary fibrosis. Patients were primarily elderly (median age, 70 years) at the time of enrollment (from 2003 to 2006). Patients were treated with pirfenidone until they discontinued the study or until the study ended in 2015. Twelve patients received pirfenidone treatment for more than 8 years. The most common side effect of pirfenidone was nausea. IPF disease progression and pneumonia were the most common serious health problems observed during the study and were not likely to be related to pirfenidone treatment. Patients who discontinued pirfenidone typically did so due to nausea or IPF disease progression. Twenty-one patients died; of these, 16 died from causes related to IPF.

## Introduction

Idiopathic pulmonary fibrosis (IPF) is a progressive, irreversible, and fatal fibrosing lung disease, with a median survival of 2–5 years after diagnosis [1–4]. Pirfenidone is an oral antifibrotic agent that slows disease progression, has anti-inflammatory properties, and was approved for the treatment of IPF in Europe in 2011 and in the United States in 2014 [5–7]. Pirfenidone has a well-characterized long-term safety profile, a favorable benefit–risk profile, and manageable tolerability [8, 9]. Evidence from postmarketing surveillance and real-world studies supports the safety profile observed in large randomized clinical trials [5, 6, 8–10].

PIPF-002 was a phase 2, multicenter, open-label study (NCT00080223) that investigated the safety of pirfenidone in patients with IPF or other types of pulmonary fibrosis (PF). Patients were enrolled from several sources, and both patients who were treatment naive and those with prior exposure to pirfenidone were included. The study provided continued access to pirfenidone until its termination in April 2015 when pirfenidone became commercially available in the United States. The study protocol was amended four times. The first two amendments modified eligibility criteria, and the third amendment updated dosing (switching patients from 400-mg capsules to 267-mg capsules) and dose modification guidelines. With the implementation of the final protocol amendment, PIPF-002 became solely a safety study; this change aimed to reduce the burden associated with the collection of efficacy data in the small number of patients remaining in the open-label study.

After the final protocol amendment, one aim of PIPF-002 was to provide continued access to pirfenidone in patients with IPF/PF. A second aim, which was the primary focus of the study, was to describe the long-term safety of pirfenidone (≤ 3600 mg/day) in the IPF/PF population. The final study results of PIPF-002 are reported here.

## Methods

### Patients

PIPF-002 was a phase 2, multicenter, open-label study of pirfenidone in patients with IPF. Between August 2003 and September 2006, patients with either IPF or other PF were enrolled in PIPF-002. From September 2005 onward, enrollment was restricted to patients with IPF. Before this restriction, “secondary PF” was listed on the case report form to broaden inclusion criteria for the study to include other types of PF. Because the focus of this study was safety, these patients were retained in the analysis.

Patients enrolled in PIPF-002 from four sources, one previous pirfenidone study and three additional sources. Patients enrolled from study PIPF-001, a double-blind comparison of pirfenidone and prednisone in patients with PF [11]. Patients also enrolled from individual-patient protocols (IPPs), investigator-sponsored investigational new drug applications (INDs), and an early access program for this study that was added with Amendment 1. The PIPF-002 study initially enrolled patients with IPF or other PF, but with Amendment 2, enrollment was restricted to patients with IPF. Patients enrolled from an IND or the early access program were treatment naive. Patients enrolled from PIPF-001 or an IPP could have received pirfenidone before the PIPF-002 study.

Because patients entered the study from four different sources, their eligibility and prior screening assessments varied. The most stringent eligibility criteria applied to patients who entered via the early access program, who had no prior exposure to pirfenidone, and had not been screened in a previous pirfenidone study. Based on the original protocol, patients were included if they met the eligibility criteria for PIPF-001, an IPP, or an IND and had adhered to the study requirements. Under Amendment 1, early access program patients aged 40–85 years were included if they had IPF symptoms for ≥ 3 months and a confirmed diagnosis of IPF (confirmed with either high-resolution computed tomography [HRCT] showing definite usual interstitial pneumonia [UIP] or surgical lung biopsy showing definite or probable UIP with HRCT confirmation of at least probable IPF) within 48 months. Patients were excluded from the early access program if they had percent predicted forced vital capacity (FVC) < 50%, hemoglobin-corrected percent predicted diffusing capacity for carbon monoxide (DLco) < 35%, resting partial pressure of arterial oxygen < 55 mmHg on room air, interstitial lung disease of known cause, a clinically significant environmental exposure known to cause PF or a connective tissue disease, significant disability, or a condition other than IPF likely to result in death within 3 years. Only patients from the early access program were enrolled under Amendments 2 and 3. In addition to the exclusion criteria detailed under Amendment 1, patients were excluded if they were enrolled in another IPF study within 60 days, had withdrawn prematurely from an IPF study within 12 months, or were eligible for another IPF study within 300 miles of their home. Enrollment criteria did not change under Amendment 4, and no patients were enrolled under this final amendment.

All procedures followed were in accordance with the ethical standards of the responsible committee on human experimentation (institutional and national) and with the Helsinki Declaration of 1964, as revised in 2013. All investigators obtained institutional review board approval for the investigation, and all patients provided informed consent. The ClinicalTrials.gov registration number for this study is NCT00080223.

### Study Design

Patients were assigned to one of three enrollment groups at study entry based on their source of enrollment, pirfenidone exposure history, and the protocol amendment as follows: group 1 comprised patients enrolled from PIPF-001 or IPP who were taking pirfenidone at enrollment or had taken the last dose ≤ 4 weeks prior; group 2 comprised patients enrolled from PIPF-001 or IPP with no prior exposure to pirfenidone or who had taken the last dose > 4 weeks before enrollment or patients enrolled from the early access program (under Amendment 1) who had no previous exposure to pirfenidone; and group 3 comprised patients enrolled from the early access program (under Amendment 2 or 3) who had no prior exposure to pirfenidone.

All patients received oral pirfenidone with food, in three divided doses daily. The dose-titration schedule and maintenance dose were determined by enrollment group (Table S1). The dose was based on body weight (40 mg/kg) and was administered via 400-mg pirfenidone capsules. In May 2006, patients were transitioned to 267-mg pirfenidone capsules, and the dose was adjusted so that the patient received a dose as close as possible to the previous dose. Patients whose maintenance dose was > 2403 mg/day continued to receive that dose as tolerated.

Dose interruptions were defined as any reported dosing gap or zero dose of study drug during the study (after the first 2 weeks of dose titration). Treatment continued until patient withdrawal or study termination.

### Safety Analyses

Adverse events (AEs) were assessed every 12 weeks and analyzed by descriptive statistics. Patients receiving dose titration were assessed more frequently during treatment initiation. Treatment-emergent AEs (TEAEs) were defined as those occurring on or after the first dosing day and ≤ 28 days after discontinuation of study treatment. The investigator judged each TEAE to be either related (“possibly related” or “probably related”) or not related to study treatment.

## Results

### Patients

A total of 83 patients at 27 sites in the United States were enrolled in PIPF-002; 70 (84.3%) were enrolled from the early access program (Table S2). A diagnosis of IPF was reported for 81 patients; other PF was reported for two patients. No patients were enrolled from an investigator-sponsored IND. Median age of patients at baseline was 70 years (range, 47–88 years), and 72.3% were male (Table 1).

**Table 1:** Demographic and baseline characteristics

Characteristic	All patients (*N* = 83)
Age, median (range), years	70 (47–88)
Male, *n* (%)	60 (72.3)
White, *n* (%)	73 (88.0)
FVC, mean (SD), percent predicted	67.7 (18.7)
DLco, mean (SD), percent predicted	38.0 (13.4)
Supplemental O_2_ use, *n* (%)	42 (50.6)
Concomitant medications of interest, *n* (%)^a^	
Prednisone	53 (63.9)
Acetylcysteine	26 (31.3)
Azathioprine	10 (12.0)
Comorbidities of interest, *n* (%)	
Gastroesophageal reflux disease	43 (51.8)
Cardiac disorders	28 (33.7)
Pulmonary hypertension	7 (8.4)
Type 2 diabetes mellitus	6 (7.2)
Emphysema	4 (4.8)
Time from IPF or PF diagnosis to first dose, median (range), years	2.7 (0.7–9.6)

*DLco* diffusing capacity for carbon monoxide, *FVC* forced vital capacity, *IPF* idiopathic pulmonary fibrosis, *O*_*2*_ oxygen, *PF* pulmonary fibrosis

^a^A total of 9 patients (10.8%) received combination therapy of prednisone, acetylcysteine, and azathioprine during the PIPF-002 study

Mean percent predicted FVC and percent predicted DLco at baseline were 67.7 and 38.0%, respectively (Table 1). Median time from IPF diagnosis was 2.7 years (range, 0.7–9.6 years). All but two patients (97.6%) had a diagnosis of IPF, and most (88.0%) had not received pirfenidone prior to study enrollment. Ten patients (12.0%) had received pirfenidone prior to this study. During the study, 53 (63.9%), 26 (31.3%), and ten patients (12.0%) received concomitant prednisone, acetylcysteine, and azathioprine, respectively. Nine patients (10.8%) received “triple therapy,” concomitant prednisone, acetylcysteine, and azathioprine, during the study.

### Pirfenidone Exposure

**Fig. 1:**
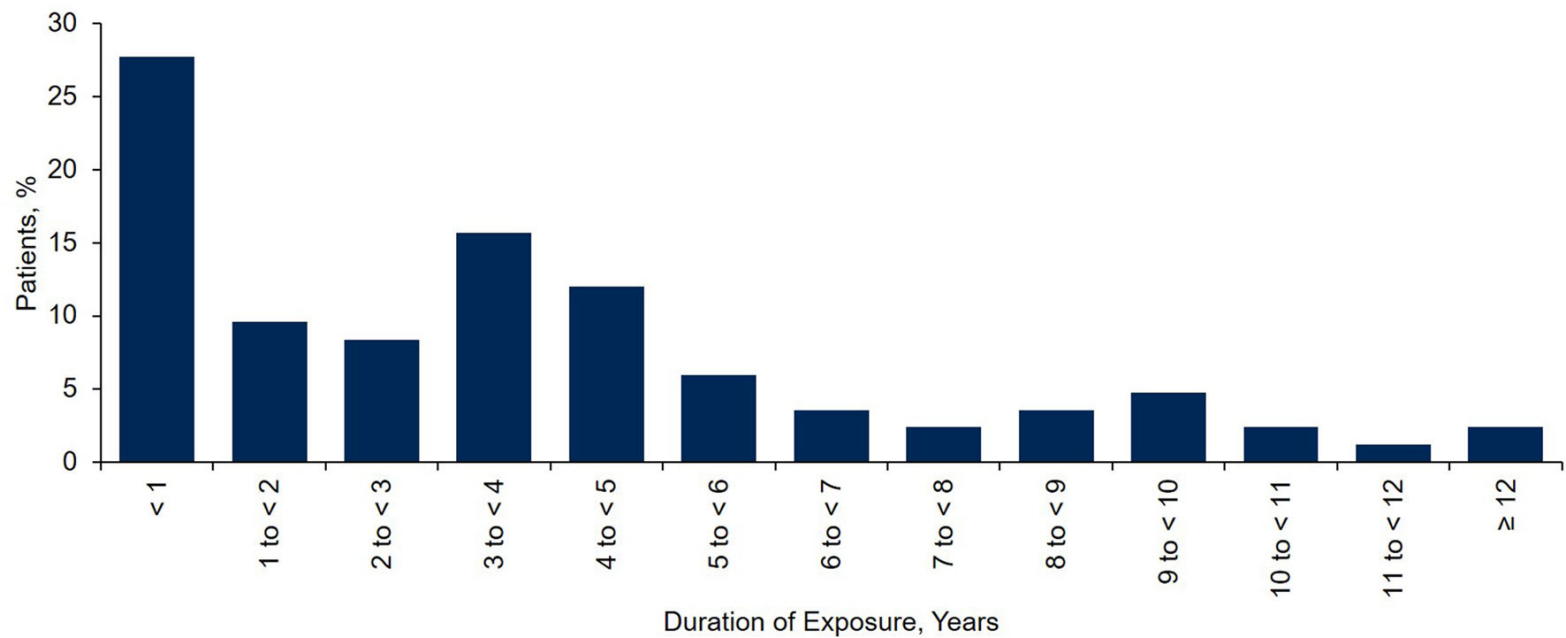
Duration of exposure to pirfenidone across studies for all patients enrolled in PIPF-002 (*N* = 83)

During PIPF-002, the median pirfenidone dose was 2400 (range, 770–3490) mg/day, with a median exposure duration of 3.0 (range, < 1–11.6) years (Table 2; Fig. 1). Because the maximum permitted dose in enrollment groups 1 and 2 was 3600 mg/day, 31 patients received ≥ 1 prescribed dose exceeding 2403 mg/day during the study.

**Table 2:** Pirfenidone exposure in study population

Pirfenidone treatment	All patients (*N* = 83)
Daily dose, median (range), mg	2400 (770–3490)
Maximum daily dose, *n* (%)^a^	
≤ 2403 mg/day	52 (62.7)
> 2403 mg/day	31 (37.3)
Duration of treatment, median (range), years^b^	
Prior to study (PIPF-001, *n* = 7; Marnac IPP, *n* = 3)	1.9 (1.3–9.1)^c^
During study (PIPF-002, *N* = 83)	3.0 (< 1–11.6)^d^
Across studies (PIPF-001, Marnac IPP, and PIPF-002, *N* = 83)	3.1 (< 1–15.6)^e^

*IPP* individual patient protocol

^a^Maximum daily dose corresponds to patients who received ≥ 1 prescribed dose > 2403 mg/day or all doses ≤ 2403 mg/day

^b^Study PIPF-001 was originally sponsored by Marnac, Inc. and completed by InterMune, Inc. Several IPPs were initiated under Marnac sponsorship. InterMune was acquired by F. Hoffmann-La Roche Ltd. in 2014

^c^The maximum duration of previous pirfenidone exposure was 9.1 years in a patient enrolled in PIPF-002 from an IPP

^d^The maximum duration of on-study pirfenidone exposure was 11.6 years in a patient who had no prior exposure to pirfenidone

^e^The maximum duration of total pirfenidone exposure was 15.6 years in a patient who had 5.6 years of exposure prior to PIPF-002 and 10.0 years of exposure during PIPF-002

Dose interruptions occurred in 29 (34.9%) patients. Median cumulative duration of dose interruption was 14.0 days (range, 1–129 days).

**Fig. 2:**
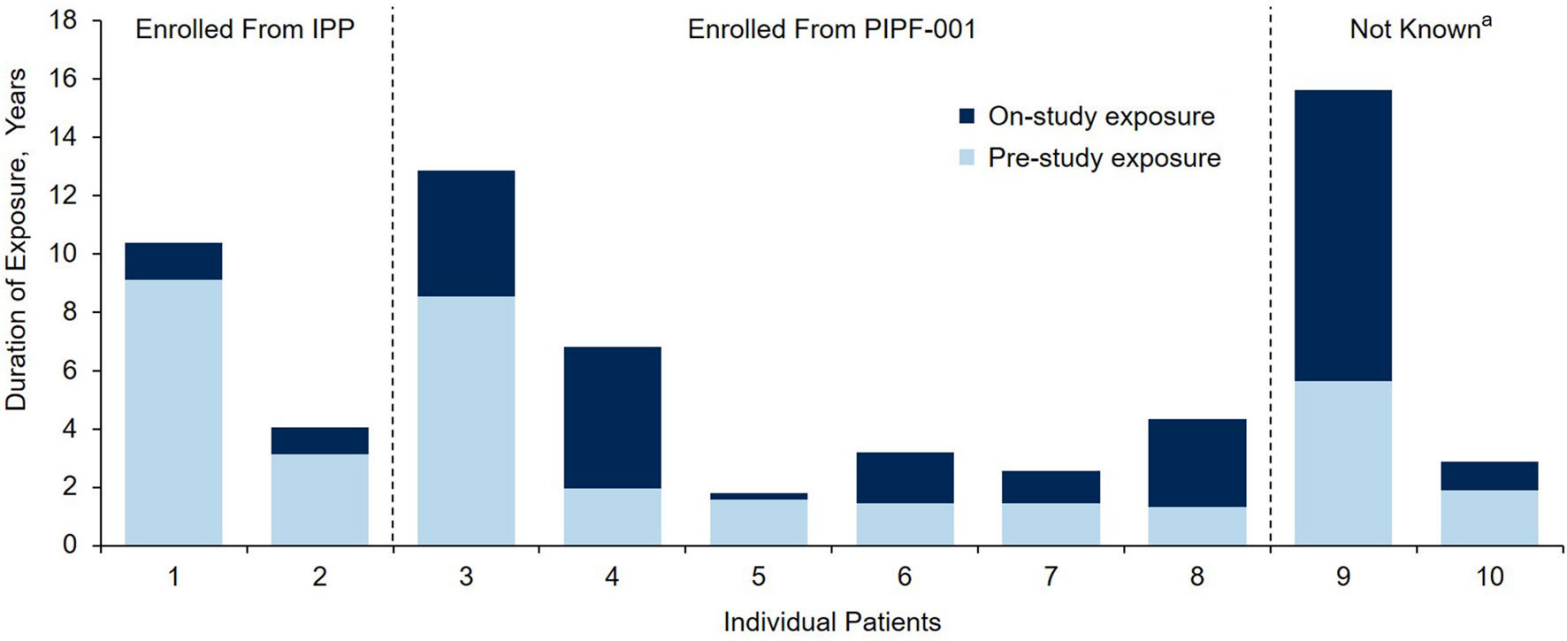
Total exposure to pirfenidone in individual patients with exposure prior to enrollment in PIPF-002 (*n* = 10), grouped by enrollment source. On-study exposure (*dark blue*) represents pirfenidone received from the time the patient enrolled in PIPF-002. Pre-study exposure (*light blue*) represents pirfenidone received during PIPF-001 or during an IPP prior to enrollment in PIPF-002. *IPP* individual patient protocol. ^a^For patient 9, the enrollment source was not recorded in the patient narrative. For patient 10, the patient narrative and enrollment source were not available in the study report

Cumulative total exposure to pirfenidone was 279.7 patient-exposure years (PEY). Twelve patients had long-term exposure to pirfenidone of ≥ 8 years (Fig. 1), including three patients with pre-study exposure. Median pre-study exposure to pirfenidone among the ten patients with prior exposure was 1.92 years (range, 1.3–9.1 years) (Table 2; Fig. 2).

### Patient Disposition

**Fig. 3:**
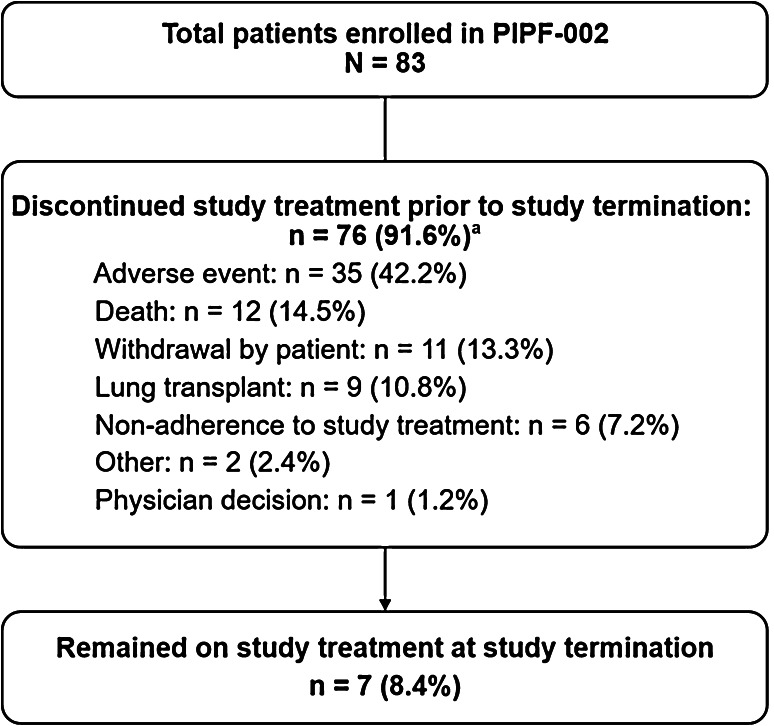
Patient disposition in PIPF-002. ^a^Until protocol Amendment 4, patients remained in the study after discontinuing pirfenidone

Seven patients (8.4%) remained in the study at the time of study termination in April 2015 and were considered to have completed the study (Fig. 3). The most common reason for pirfenidone discontinuation was TEAEs (35 patients [12.5 per 100 PEY]); these included IPF progression (five patients [1.8 per 100 PEY]) and nausea (four patients [1.4 per 100 PEY]).

### Safety

Most patients reported ≥ 1 TEAE (98.8%), with 41.0% of patients reporting mild to moderate TEAEs, 36.1% reporting severe TEAEs, and 21.7% reporting life-threatening TEAEs (Table 3). The most commonly reported severe TEAEs were IPF progression (10.8%), pneumonia and dyspnea (3.6% each), and coronary artery disease, nausea, and pneumothorax (2.4% each). IPF progression was defined as any TEAE that was reported as IPF as judged by the investigator. The most commonly reported life-threatening TEAEs were IPF and respiratory failure (6.0% each) and pneumonia (2.4%).

**Table 3:** Overview of TEAEs and serious TEAEs

TEAE, *n* (%)	Pirfenidone ≤ 2403 mg/day (*n* = 52)^a^	Pirfenidone > 2403 mg/day (*n* = 31)^a^	All patients (*N* = 83)
Patients with ≥ 1 TEAE	52 (100.0)	30 (96.8)	82 (98.8)
Patients with TEAEs by maximum intensity^b^			
Mild	4 (7.7)	0	4 (4.8)
Moderate	21 (40.4)	9 (29.0)	30 (36.1)
Severe	16 (30.8)	14 (45.2)	30 (36.1)
Life-threatening	11 (21.2)	7 (22.6)	18 (21.7)
TEAEs by relationship to pirfenidone^c^			
Unrelated	6 (11.5)	5 (16.1)	11 (13.3)
Possibly related	26 (50.0)	5 (16.1)	31 (37.3)
Probably related	20 (38.5)	20 (64.5)	40 (48.2)
≥ 1 serious TEAE	32 (61.5)	17 (54.8)	49 (59.0)
≥ 1 treatment-related serious TEAE^d^	6 (11.5)	3 (9.7)	9 (10.8)
Discontinuation of pirfenidone due to TEAE	28 (53.8)	8 (25.8)	36 (43.4)^e^
Study withdrawal due to TEAE	30 (57.7)	13 (41.9)	43 (51.8)
Death as an outcome of TEAE	12 (23.1)	9 (29.0)	21 (25.3)^f^

*TEAE* treatment-emergent adverse event

^a^Patients were categorized by maximum daily dose received at any time (≥ 1 prescribed dose > 2403 mg/day or all doses ≤ 2403 mg/day)

^b^Patients experiencing TEAEs of more than one intensity are summarized according to the maximum intensity experienced for all TEAEs

^c^Each patient was counted only once at the strongest relationship of any event for that patient

^d^A TEAE is considered related to study treatment if the investigator indicated it was possibly or probably related to study treatment

^e^Due to a data entry error, one patient was counted as “discontinued due to a TEAE” in one data source and as “discontinued study treatment due to death” in another

^f^One additional patient died > 28 days after the last dose due to a non-TEAE

Overall, there were 460.5 TEAEs per 100 PEY (Table 4). The most common TEAEs were nausea (23.6 per 100 PEY), IPF progression (16.1 per 100 PEY), fatigue (11.8 per 100 PEY), dyspnea (11.4 per 100 PEY), upper respiratory tract infection (11.4 per 100 PEY), and cough (10.7 per 100 PEY).

**Table 4:** Incidence rates of TEAEs occurring in ≥ 10% of patients and other TEAEs of interest

TEAE by preferred term	Patients with ≥ 1 event,* n* (%) (*N* = 83)	Events, *n*	Adjusted incidence per 100 PEY^a^
TEAEs occurring in ≥ 10% of patients	82 (98.8)	1288	460.5
Nausea	40 (48.2)	66	23.6
IPF	29 (34.9)	45	16.1
Fatigue	27 (32.5)	33	11.8
Dyspnea	25 (30.1)	32	11.4
Upper respiratory tract infection	21 (25.3)	32	11.4
Cough	21 (25.3)	30	10.7
Rash	16 (19.3)	22	7.9
Weight decreased	18 (21.7)	20	7.2
Vomiting	13 (15.7)	20	7.2
Bronchitis	12 (14.5)	19	6.8
Urinary tract infection	11 (13.3)	19	6.8
Insomnia	15 (18.1)	18	6.4
Headache	14 (16.9)	17	6.1
Appetite decreased	14 (16.9)	16	5.7
Sinusitis	11 (13.3)	15	5.4
Depression	12 (14.5)	14	5.0
Anorexia	11 (13.3)	14	5.0
Dizziness	12 (14.5)	13	4.6
Pneumonia	10 (12.0)	13	4.6
Diarrhea	10 (12.0)	13	4.6
Back pain	10 (12.0)	13	4.6
Anxiety	10 (12.0)	13	4.6
Constipation	10 (12.0)	12	4.3
Gastroesophageal reflux disease	10 (12.0)	12	4.3
Pulmonary hypertension	10 (12.0)	10	3.6
Peripheral edema	9 (10.8)	9	3.2
Other TEAEs of interest			
Respiratory tract infection	6 (7.2)	11	3.9
Photosensitivity reaction	6 (7.2)	10	3.6
Abdominal discomfort	7 (8.4)	9	3.2
Respiratory failure	6 (7.2)	8	2.9
Dyspepsia	7 (8.4)	7	2.5
Stomach discomfort	5 (6.0)	6	2.1

*IPF* idiopathic pulmonary fibrosis, *PEY* patient exposure years, *TEAE* treatment-emergent adverse event

^a^Adjusted incidence = number of events/total exposure in PEY × 100. The total exposure was 279.7 PEY

The most common treatment-related TEAEs were nausea (14.7 per 100 PEY), fatigue (10.0 per 100 PEY), weight decreased (6.1 per 100 PEY), rash (5.0 per 100 PEY), and appetite decreased (4.6 per 100 PEY). Nausea occurred in similar proportions of patients who received pirfenidone at a maximum dose > 2403 mg/day and those at ≤ 2403 mg/day; in contrast, fatigue, rash, and photosensitivity reactions occurred in a greater proportion of patients who received pirfenidone at a maximum dose > 2403 mg/day (Table S3). Among the nine patients who received concomitant prednisone, acetylcysteine, and azathioprine (“triple therapy”), the most frequently reported TEAEs were nausea and dyspnea (four patients each) followed by respiratory failure, fatigue, decreased weight, and stomach discomfort (three patients each).

Forty-nine patients (59.0% [116 events for 41.5 per 100 PEY]) had ≥ 1 serious TEAE (Table 3). The most common serious TEAEs were IPF progression (18.1% [16 events for 5.7 per 100 PEY]), pneumonia (10.8% [11 events for 3.9 per 100 PEY]), and respiratory failure (7.2% [six events for 2.1 per 100 PEY]). TEAEs leading to permanent discontinuation of pirfenidone occurred in 36 patients (43.4% [46 events for 16.4 per 100 PEY]); those leading to study withdrawal occurred in 43 patients (51.8% [48 events for 17.2 per 100 PEY]; Table 3). TEAEs that led to study drug discontinuation included IPF in five patients (6.0% [five events for 1.8 per 100 PEY]), nausea in four patients (4.8% [four events for 1.4 per 100 PEY]), and weight decreased in 3 patients (3.6% [three events for 1.1 per 100 PEY]).

**Fig. 4:**
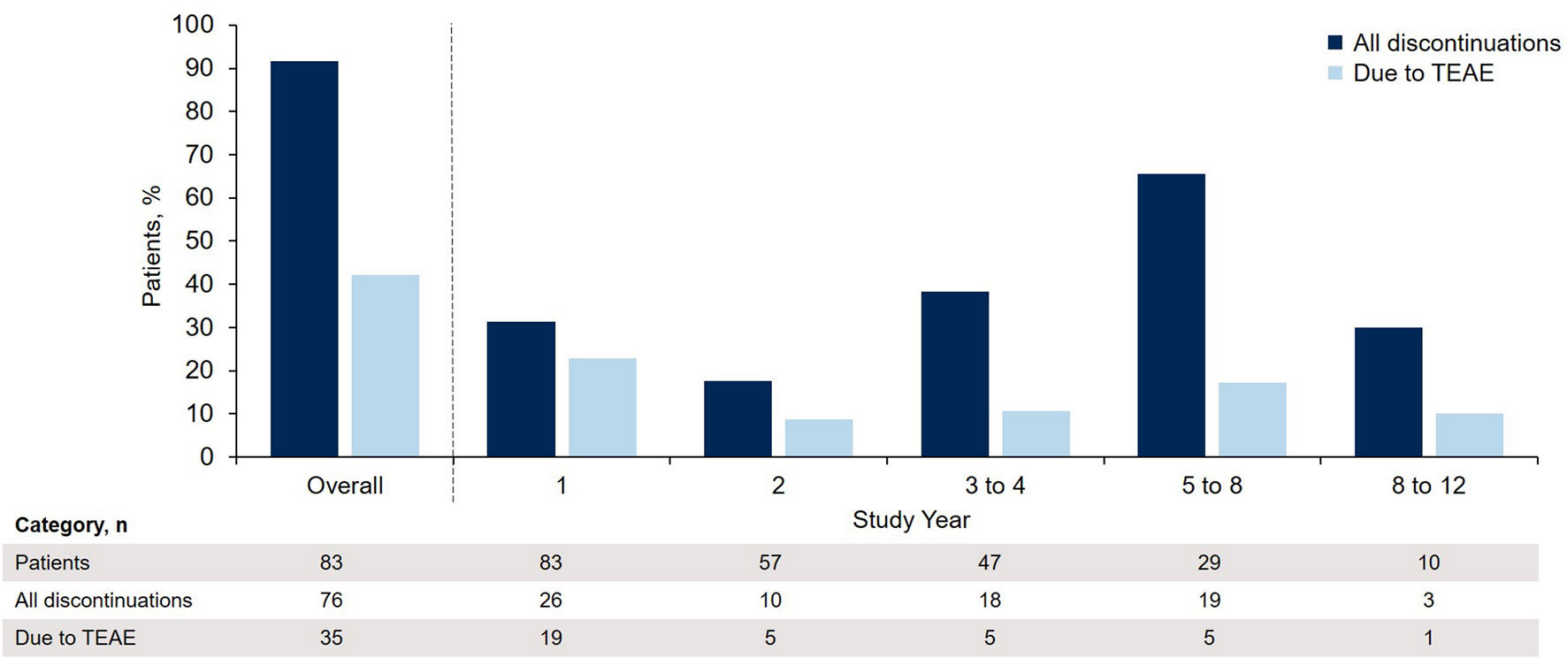
Total pirfenidone discontinuations over time in PIPF-002. Patient percentages are based on the number of patients who participated during the given time period. All discontinuations (*dark blue*) and discontinuations due to TEAE (*light blue*) within each time period are shown. *TEAE* treatment-emergent adverse event

The proportion of discontinuations due to TEAEs relative to the total number of discontinuations in any given period generally decreased over the course of the study and was highest in study year 1 (22.9%; Fig. 4). From year 2, the main reasons for pirfenidone discontinuation were withdrawal by patient, death, and lung transplant; the latter two reasons reflect the natural history of the disease.

Twenty-one patients (7.5 per 100 PEY) died within 28 days of their last dose of pirfenidone; 16 deaths (5.7 per 100 PEY) were related to IPF. The five deaths not related to IPF were due to ventricular fibrillation, starvation, metastatic neoplasm, non-small cell lung cancer, and pulmonary embolism (one each). The two deaths assessed by the investigator as possibly related to study treatment were due to respiratory failure (study day 635) and IPF (study day 185).

## Discussion

The results of the PIPF-002 study form an early description of the long-term safety profile of pirfenidone, extending up to approximately 12 years, in patients with IPF (97.6%) and other non-IPF fibrotic interstitial lung diseases (2.4%). Long-term exposure to pirfenidone (median duration, 3.0 years) was found to be generally safe and well tolerated in this patient population. TEAEs observed during the study were consistent with those expected in an elderly population of patients with IPF and with the known safety profile of pirfenidone. During the study, 25.3% of patients died, and most deaths were IPF-related. This rate and pattern of deaths was reflective of the natural history of IPF and the age of the patient population [3].

Many TEAEs in PIPF-002 were related to tolerability and were manageable through dose modification, as evidenced by comparing the TEAEs listed as reasons for discontinuation with the frequency with which these TEAEs were reported. Nausea and decreased weight were reported in 40 and 18 patients, respectively, but these TEAEs accounted for only 4 and 3 discontinuations, respectively. Dose modifications are an effective strategy for managing TEAEs associated with pirfenidone, particularly gastrointestinal- and skin-related TEAEs [12]. Importantly, in a pooled analysis of data from the phase 3 ASCEND (study 016; NCT01366209) and CAPACITY (studies 004 and 006; NCT002387716 and NCT00287729) trials, the efficacy of pirfenidone was maintained in patients who underwent dose reductions or dose interruptions [13]. PIPF-002 included 12 patients on long-term pirfenidone therapy (> 8 years), with no clear safety signals observed for this duration. The exposure duration in PIPF-002 was longer than in RECAP (NCT00662038), an open-label long-term extension study of the ASCEND and CAPACITY trials [14]. In RECAP, patients had a median duration of pirfenidone exposure of 1.7 years (maximum, 6.1 years) [14].

This study had several limitations. Due to design limitations of a long-term open-label study with a single treatment arm, no conclusions can be made regarding the potential stability of pulmonary function related to treatment with pirfenidone. A group of patients in this study received pirfenidone at a dose higher than the currently recommended daily dose and higher than that used in the phase 3 clinical trials; therefore, a potential dose–response for study outcomes should be considered in line with the known safety profile of pirfenidone. A subgroup of patients had pre-study exposure to pirfenidone with variable durations; this subgroup likely selected against patients with tolerability issues. The study protocol was amended several times, and the study had a relatively small enrollment population. Concomitant corticosteroid use was different in the PIPF-002 study population compared with the phase 3 clinical trial populations. At baseline in this study, > 60% of patients were receiving concomitant prednisone and 12% were receiving concomitant azathioprine. In contrast, concomitant corticosteroid use was not permitted in CAPACITY and was permitted for ≤ 28 days to treat an acute exacerbation in ASCEND, while azathioprine was permitted only for short courses following an acute exacerbation or disease progression event in CAPACITY and was not permitted in ASCEND [5, 6]. In past years, patients with IPF were frequently treated with corticosteroids despite a lack of evidence for efficacy or with azathioprine despite weak evidence for efficacy; current treatment guidelines do not recommend these treatments for most patients with IPF [1, 7, 15]. These differences in concomitant medication use could have affected the incidence of observed TEAEs in PIPF-002 compared with those in ASCEND and CAPACITY. Finally, 37% of patients received pirfenidone at higher doses than the approved indication (> 2403 mg/day). Thus, the TEAE profile observed in PIPF-002 may not fully reflect the true safety profile of pirfenidone alone and at the approved dose.

## Conclusions

A favorable long-term safety experience with pirfenidone over a median duration of 3.0 years, with a maximum exposure of 11.6 years, was observed during this study. Twelve patients received pirfenidone for > 8 years. Findings were generally consistent with the progressive nature of the underlying disease and with the known safety profile of pirfenidone, with no new safety signals identified. This study provides additional long-term safety data to support the continued use of pirfenidone in patients with IPF.

### Electronic supplementary material

Below is the link to the electronic supplementary material.

Supplementary material 1 (PDF 250 kb)

## References

[1] Raghu G, Collard HR, Egan JJ (2011). An official ATS/ERS/JRS/ALAT statement: idiopathic pulmonary fibrosis: evidence-based guidelines for diagnosis and management. Am J Respir Crit Care Med.

[2] Fernandez Perez ER, Daniels CE, Schroeder DR (2010). Incidence, prevalence, and clinical course of idiopathic pulmonary fibrosis: a population-based study. Chest.

[3] Ley B, Collard HR, King TE (2011). Clinical course and prediction of survival in idiopathic pulmonary fibrosis. Am J Respir Crit Care Med.

[4] Nathan SD, Shlobin OA, Weir N (2011). Long-term course and prognosis of idiopathic pulmonary fibrosis in the new millennium. Chest.

[5] Noble PW, Albera C, Bradford WZ (2011). Pirfenidone in patients with idiopathic pulmonary fibrosis (CAPACITY): two randomised trials. Lancet.

[6] King TE, Bradford WZ, Castro-Bernardini S (2014). A phase 3 trial of pirfenidone in patients with idiopathic pulmonary fibrosis. N Engl J Med.

[7] Raghu G, Rochwerg B, Zhang Y (2015). An official ATS/ERS/JRS/ALAT clinical practice guideline: treatment of idiopathic pulmonary fibrosis. An update of the 2011 clinical practice guideline. Am J Respir Crit Care Med.

[8] Lancaster L, Albera C, Bradford WZ (2016). Safety of pirfenidone in patients with idiopathic pulmonary fibrosis: integrated analysis of cumulative data from 5 clinical trials. BMJ Open Respir Res.

[9] Valeyre D, Albera C, Bradford WZ (2014). Comprehensive assessment of the long-term safety of pirfenidone in patients with idiopathic pulmonary fibrosis. Respirology.

[10] Chaudhuri N, Duck A, Frank R, Holme J, Leonard C (2014). Real world experiences: pirfenidone is well tolerated in patients with idiopathic pulmonary fibrosis. Respir Med.

[11] Moises S, Girod CE, Shifren A (2003). A double-blind, multicenter study comparing pirfenidone and prednisone for moderate-to-severe pulmonary fibrosis. Chest.

[12] Lancaster LH, de Andrade JA, Zibrak JD (2017). Pirfenidone safety and adverse event management in idiopathic pulmonary fibrosis. Eur Respir Rev.

[13] Nathan SD, Lancaster LH, Albera C (2016). Dose modifications and dose intensity during treatment with pirfenidone (Abstract). Eur Respir J.

[14] Costabel U, Albera C, Lancaster LH (2017). An open-label study of the long-term safety of pirfenidone in patients with idiopathic pulmonary fibrosis (RECAP). Respiration.

[15] Richeldi L, Davies HR, Ferrara G, Franco F (2003). Corticosteroids for idiopathic pulmonary fibrosis. Cochrane Database Syst Rev.

