# Supplementary material for: An Open-Label, Phase II Study of the Safety of Pirfenidone in Patients with Idiopathic Pulmonary Fibrosis (PIPF-002)
Source: Pulm Ther. 2018 Jun 1;4(1):59–71. doi: 10.1007/s41030-018-0053-y (PMC6967037; doi:10.1007/s41030-018-0053-y)
Supplement: Supplementary file 1 — Supplementary material 1 (PDF 250 kb) [file 41030_2018_53_MOESM1_ESM.pdf]

An Open-Label, Phase II Study of the Safety of Pirfenidone in Patients with Idiopathic Pulmonary Fibrosis (PIPF-002)

SUPPLEMENTARY MATERIAL

This supplement contains 3 tables.

Table S1. Dose titration and dosing by enrollment group

**Patient enrollment group**[a]	**Pirfenidone dose (as tolerated)**[b]	**Initial dose titration**
Group 1 (*n* = 4)	Started study treatment at prior dose (maximum, 3600 mg/day)	• Not required
Group 2 (*n* = 37)	Target maintenance dose:<br>• 40 mg/kg (maximum, 3600 mg/day)	• Dose was titrated over 10 to 14 days to target maintenance dose, based on body weight and tolerability
Group 3 (*n* = 42)	Target maintenance dose:<br>• With 400-mg capsules, 2400 mg/day<br>• With 267-mg capsules, 2403 mg/day	• With 400-mg capsules, dose was titrated over 10 days to target maintenance dose, as tolerated<br>• With 267-mg capsules, dose was titrated over 15 days to target maintenance dose, as tolerated

[a] Patients in group 1 were receiving pirfenidone at enrollment or had received their last dose ≤ 4 weeks before enrollment. Patients in group 2 had no previous exposure to pirfenidone or received their last dose > 4 weeks before enrollment. Patients in group 3 had no previous exposure to pirfenidone and enrolled after implementation of protocol Amendment 2.

[b] With the implementation of Amendment 3, patients were transitioned from 400-mg to 267-mg capsules, and the dose was adjusted to be as close as possible to the previous dose without exceeding the maximum permitted dose. Patients whose maintenance dose was > 2403 mg/day (≤ 3600 mg/day) continued to receive that dose.

Table S2. Enrollment by patient source, all treated patients

**Patients by enrollment source, *n*[a]**	**Patient enrollment groups[b]**			**All patients (*N* = 83)**
	**Group 1 (*n* = 4)**	**Group 2 (*n* = 37)**	**Group 3 (*n* = 42)**	
Study PIPF-001	1	9	0	10
Prior pirfenidone	1	6	0	7
Marnac-sponsored IPP	3	0	0	3
Prior pirfenidone	3	0	0	3
Investigator-sponsored IND	0	0	0	0
Early access program	0	28	42	70

IND, investigational new drug; IPP, individual patient protocol.

[a] Study PIPF-001 was originally sponsored by Marnac and completed by InterMune. Several IPPs were initiated under Marnac sponsorship. InterMune was acquired by F. Hoffmann-La Roche in 2014.

[b] Patients in group 1 were receiving pirfenidone at enrollment or had received their last dose ≤ 4 weeks before enrollment. Patients in group 2 had no previous exposure to pirfenidone or received their last dose > 4 weeks before enrollment. Patients in group 3 had no previous exposure to pirfenidone and enrolled after implementation of protocol Amendment 2.

Table S3. TEAEs occurring in ≥ 5% of patients

Patients with ≥ 1 TEAE by preferred term, *n* (%)	Pirfenidone ≤ 2403 mg/day (*n* = 52)[a]	Pirfenidone > 2403 mg/day (*n* = 31)[a]	All patients (*N* = 83)
**All TEAEs**	**52 (100.0)**	**30 (96.8)**	**82 (98.8)**
Nausea	25 (48.1)	15 (48.4)	40 (48.2)
IPF	18 (34.6)	11 (35.5)	29 (34.9)
Fatigue	15 (28.8)	12 (38.7)	27 (32.5)
Dyspnea	18 (34.6)	7 (22.6)	25 (30.1)
Upper respiratory tract infection	13 (25.0)	8 (25.8)	21 (25.3)
Cough	11 (21.2)	10 (32.3)	21 (25.3)
Weight decreased	11 (21.2)	7 (22.6)	18 (21.7)
Rash	7 (13.5)	9 (29.0)	16 (19.3)
Insomnia	11 (21.2)	4 (12.9)	15 (18.1)
Headache	7 (13.5)	7 (22.6)	14 (16.9)
Appetite decreased	8 (15.4)	6 (19.4)	14 (16.9)
Vomiting	10 (19.2)	3 (9.7)	13 (15.7)
Bronchitis	9 (17.3)	3 (9.7)	12 (14.5)
Depression	7 (13.5)	5 (16.1)	12 (14.5)
Dizziness	10 (19.2)	2 (6.5)	12 (14.5)
Urinary tract infection	8 (15.4)	3 (9.7)	11 (13.3)
Sinusitis	7 (13.5)	4 (12.9)	11 (13.3)
Anorexia	8 (15.4)	3 (9.7)	11 (13.3)
Pneumonia	6 (11.5)	4 (12.9)	10 (12.0)
Diarrhea	6 (11.5)	4 (12.9)	10 (12.0)
Back pain	7 (13.5)	3 (9.7)	10 (12.0)
Anxiety	5 (9.6)	5 (16.1)	10 (12.0)
Constipation	7 (13.5)	3 (9.7)	10 (12.0)
Gastroesophageal reflux disease	5 (9.6)	5 (16.1)	10 (12.0)
Pulmonary hypertension	8 (15.4)	2 (6.5)	10 (12.0)
Peripheral edema	4 (7.7)	5 (16.1)	9 (10.8)
Asthenia	7 (13.5)	1 (3.2)	8 (9.6)
Hypertension	6 (11.5)	2 (6.5)	8 (9.6)
Nasal congestion	5 (9.6)	3 (9.7)	8 (9.6)
Nasopharyngitis	5 (9.6)	3 (9.7)	8 (9.6)
Abdominal discomfort	6 (11.5)	1 (3.2	7 (8.4)
Dyspepsia	5 (9.6)	2 (6.5)	7 (8.4)
Pyrexia	5 (9.6)	2 (6.5)	7 (8.4)
Anemia	3 (5.8)	3 (9.7)	6 (7.2)
Contusion	6 (11.5)	0	6 (7.2)
Myalgia	5 (9.6)	1 (3.2)	6 (7.2)
Photosensitivity reaction	1 (1.9)	5 (16.1)	6 (7.2)
Respiratory failure	5 (9.6)	1 (3.2)	6 (7.2)
Respiratory tract infection	4 (7.7)	2 (6.5)	6 (7.2)
Skin laceration	5 (9.6)	1 (3.2)	6 (7.2)
Coronary artery disease	3 (5.8)	2 (6.5)	5 (6.0)
Proteinuria	4 (7.7)	1 (3.2)	5 (6.0)
Pruritus	1 (1.9)	4 (12.9)	5 (6.0)
Stomach discomfort	4 (7.7)	1 (3.2)	5 (6.0)

IPF, idiopathic pulmonary fibrosis; TEAE, treatment-emergent adverse event.

[a] Patients were categorized by maximum daily dose received at any time (≥ 1 prescribed dose > 2403 mg/day or all doses ≤ 2403 mg/day).
